# Resurgence of malaria in the Amhara Region, Ethiopia (2014–2024): trends, spatial expansion, and control challenges

**DOI:** 10.1186/s12936-025-05668-0

**Published:** 2025-11-25

**Authors:** Mastewal Worku Lake, Mulusew Andualem Asemahagn, Teshager Zerihun Nigussie, Kassahun Alemu Gelaye, Kindie Fentahun Muchie, Hailemariam Awoke Engedaw, Muluken Azage Yenesew

**Affiliations:** 1 Amhara Public Health Institute, Bahir Dar, Ethiopia; 2 Department of Epidemiology and Biostatistics, School of Public Health, College of Medicine and Health Sciences, Bahir Dar University, Bahir Dar, Ethiopia; 3 Department of Health System and Health Economics, School of Public Health, College of Medicine and Health Sciences, Bahir Dar University, Bahir Dar, Ethiopia; 4 Department of Statistics, College of Natural and Computational Sciences, Debre Tabor University, Debre Tabor, Ethiopia; 5 Department of Epidemiology and Biostatistics, Institute of Public Health, College of Medicine and Health Sciences, University of Gondar, Gondar, Ethiopia; 6 Department for Infectious Disease and Tropical Medicine, University Hospital Heidelberg, Heidelberg, Germany; 7 Department of Internal Medicine, School of Medicine, College of Medicine and Health Sciences, Bahir Dar University, Bahir Dar, Ethiopia; 8 Department of Environmental Health, School of Public Health, College of Medicine and Health Sciences, Bahir Dar University, Bahir Dar, Ethiopia

**Keywords:** Malaria resurgence, *Plasmodium vivax*, Epidemiological transition, Spatial epidemiology, Transmission hotspots, Ethiopia, Elimination

## Abstract

**Background:**

Despite substantial control gains over the past 2 decades, malaria remains a major public health threat in Ethiopia. The Amhara Region has recently experienced a significant resurgence, threatening to reverse previous progress. However, comprehensive analyses of this resurgence integrating long-term trends, parasite species dynamics, and spatiotemporal patterns are limited. Therefore, this study examines trends, spatial expansion, and control challenges associated with malaria resurgence in the Amhara Region.

**Methods:**

A retrospective analysis of 11 years (January 2014–December 2024) of weekly malaria surveillance data from 166 districts in the Amhara Region, Ethiopia. Joinpoint regression was employed to detect significant temporal trend changes. A resurgence threshold was defined a priori as a ≥ 20% increase in cases compared to the 3-year average baseline for the same period (Sep–Dec 2021–2023), based on an expert consensus survey. Auto-regressive integrated moving average modelling was used to characterize and forecast the test positivity rate (TPR). The Getis-Ord Gi* statistic was used to identify spatial clustering and detect transmission hotspots.

**Results:**

During the study period, 7,710,733 malaria cases and 162 deaths were reported. Adults (≥ 15 years) contributed 62% of cases. A single trend inflection point occurred in late 2018, marking a shift from a significant decline (Annual Percent Change [APC]: −13.2%) to a sharp resurgence (APC: + 12.6%, 2018–2024). The Annual Parasite Incidence (API) rose from 10.9 (95% CI 10.8–11.0) per 1000 in 2018 to 74.8 (95% CI 74.72–74.93) per 1000 in 2024. *Plasmodium vivax* became increasingly prominent, with its contribution to the total case burden rising from 25 to 43% (χ^2^ = 190,789.55, p < 0.001). Its incidence surged 11-fold between 2018 and 2024 (from 2.7 to 32.4 per 1000). TPR increased from 10% (2018) to > 50% during peak months by 2024. In 2024, a resurgence occurred in 83% of districts; hotspots expanded into urban centres and previously low-transmission highland areas.

**Conclusion:**

The Amhara Region is experiencing a malaria resurgence characterized by intensified transmission, geographic expansion into urban and highland areas, and a significant shift toward *Plasmodium vivax* dominance. These findings exemplify an "elimination-resurgence paradox," where prior success increases vulnerability to threats such as invasive vectors, conflict, and climate shifts.

**Supplementary Information:**

The online version contains supplementary material available at 10.1186/s12936-025-05668-0.

## Background

Malaria remains a major public health problem worldwide, with an estimated 263 million cases and 597,000 deaths (mortality rate of 13.7 per 100,000) reported in 2023. According to the World Malaria Report 2024, the WHO African Region accounts for 94% of cases and 95% of malaria deaths [1]. Ethiopia ranks among the 15 countries with the highest malaria burden, contributing 3.6% of global cases, 3.1% of deaths, and 15% of cases in East and Southern Africa in 2023 [2, 3]. Nearly 69% of its population is at risk due to widespread endemicity across 75% of the country’s landmass [4].

In Ethiopia, the Amhara Region remains a persistent malaria hotspot due to its diverse altitudinal and ecological gradients. It has historically contributed a large part of the national malaria burden [5, 6], accounting for 18% of all confirmed malaria cases nationally in 2024 [3]. Substantial declines between 2004 and 2018, through scaled-up interventions including insecticide-treated nets (ITNs), indoor residual spraying (IRS), and artemisinin-based combination therapy (ACT), supported the launch of a subnational elimination strategy in 2017 and its expansion nationally by 2021 [7]. However, since 2018, surveillance data indicate a marked resurgence in transmission, threatening to reverse these gains [8].

This pattern aligns with what may be termed an “elimination resurgence paradox”: prior success may reduce operational vigilance and obscure systemic vulnerabilities, increasing susceptibility to emerging threats. Multiple drivers likely underpin the resurgence: expansion of *Anopheles stephensi* in urban settings [9–14], evolving insecticide and artemisinin resistance [5, 15, 16], climate variability [17, 18], and programmatic disruptions [19, 20], including during COVID-19 [21, 22] and conflicts, which have severely hampered vector control activities like IRS and ITN distribution [12], evidence indicates that the risk of relapsing *Plasmodium vivax* malaria is raised following an acute *Plasmodium falciparum* infection [23–26], a challenge given *P. vivax* capacity to form dormant liver hypnozoites that trigger relapses [27]. Collectively, these drivers have weakened vector control and case management, leaving populations more vulnerable.

While national surveillance data confirm a concerning rise in malaria incidence across Ethiopia, including the Amhara Region [3, 8], no prior study has mapped district-level resurgence dynamics across the region with species distribution, spatial clustering, and temporal forecasting. Although localized studies have begun to explore transmission patterns, species shifts, and spatial changes [28–31], a granular, high-resolution understanding of the resurgence across all districts in the Amhara region is critically limited.

Despite the progress made towards controlling malaria, its resurgence in combination with emerging issues highlights a need for adaptable strategies and enhanced surveillance mechanisms in order to sustain control efforts. This study quantifies the decade-long epidemiological trends, maps the spatiotemporal expansion of transmission, and identifies control challenges across all districts in the Amhara Region from January 2014 to December 2024. Findings to help guide targeted interventions and support Ethiopia’s national malaria elimination goals.

## Methods

### Study design and period

A retrospective time-series study was conducted using routine weekly malaria surveillance data from January 1, 2014, to December 30, 2024. Weeks were assigned to months based on the ISO 8601 convention (if ≥ 4 days fell within that month). The unit of analysis was the district-week (166 districts × 52 weeks/year × 11 years = 94,832 observations). Primary outcomes were: Annual parasite incidence (API): confirmed malaria cases per 1000 population, Test positivity rate (TPR): confirmed positive tests per 100 tests performed [32].

### Study setting

The study was conducted in the Amhara Region of Ethiopia (9°20′–14°20′N; 36°20′–40°20′E). Based on 2007 census projections, the region has a population above 26 million people [33] and bears a high malaria burden. Elevation levels were assigned using the NASA SRTMGL1 v3 (30 m) digital elevation model, aggregated to district median altitude: highland (> 2300 m), midland (1500–2300 m), and lowland (< 1500 m) [34]. Its monsoonal climate features a dry season (December–March) and a rainy season (April–August, peaking in June–August) [35].

**Fig. 1:**
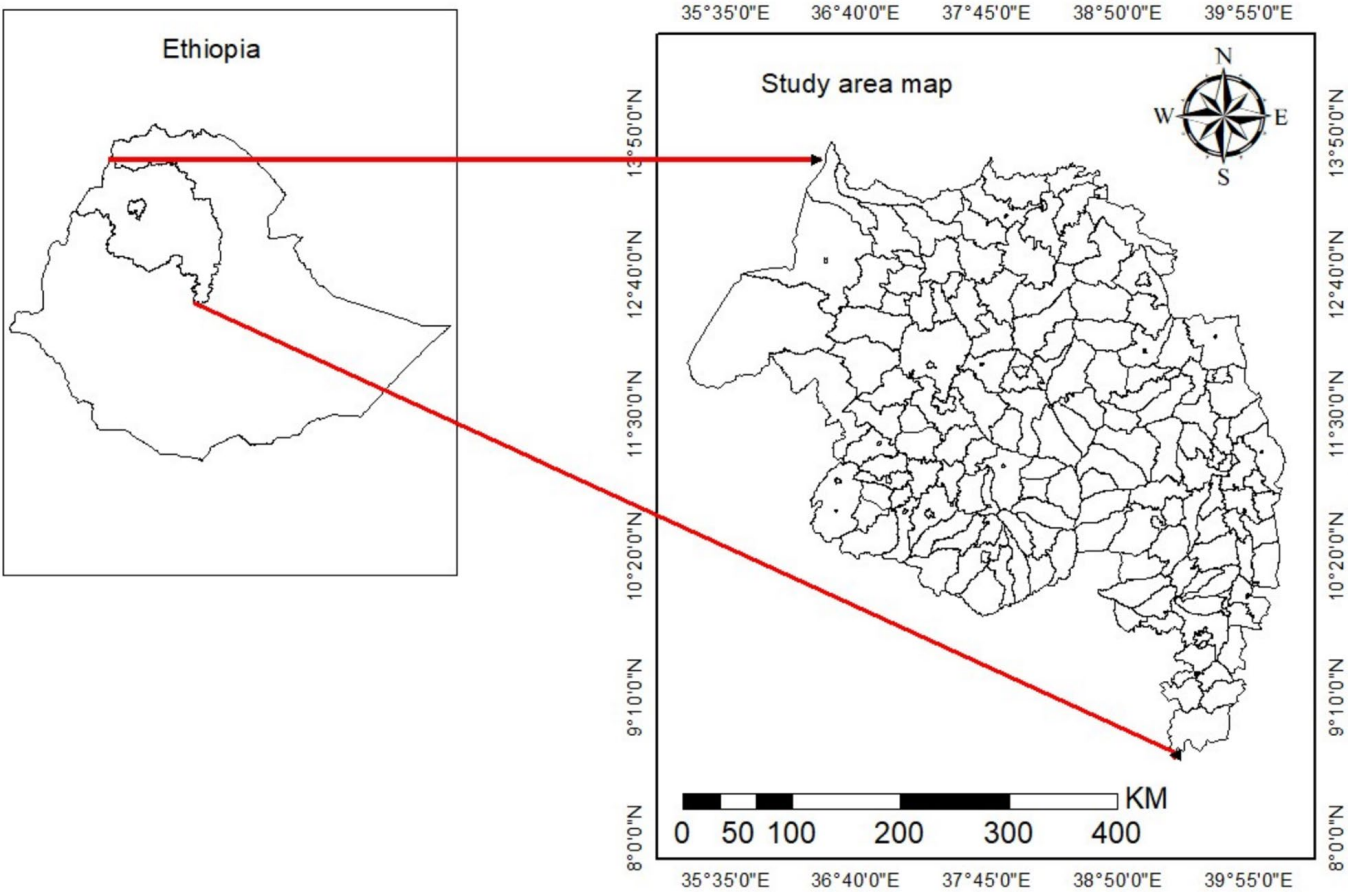
Study area map, 166 districts in the Amhara region that report weekly malaria surveillance data from June 2014 to 2024

Administratively, the region comprises 22 zones and 236 districts. Of the 236 districts, 166 were included in the analysis, as they provided consistent weekly reporting throughout the study period. The remaining districts reported data aggregated under parent administrative units due to missingness but structural reporting constraints. These 166 districts capture > 90% of regional transmission and ensure analytical representativeness (Fig. 1).

### Inclusion and exclusion criteria

All confirmed malaria cases from public and private facilities (health posts, centers, hospitals, clinics) within the 166 districts were included. Cases reported from referral and specialized hospitals were excluded to avoid overestimation due to patient inflow from non-resident populations.

### Data sources and variables

Surveillance data were obtained from the Amhara Public Health Institute's (APHI's) Public Health Emergency Management (PHEM) weekly reports (January 2014–December 2024). Variables included district identifiers, epidemiological week and year, confirmed malaria cases, tests performed (microscopy or RDT), *Plasmodium* species (*P. falciparum, P. vivax*), age group (< 5, 5–14, ≥ 15 years), and TPR. Where available, RDT type (Pf-only HRP2, Pf/Pan, Pf/Pv) was extracted to support species-trend sensitivity analyses. Population denominators were derived from the CSA intercensal projections [33]. Monthly district populations were linearly interpolated from annual estimates; age-specific denominators were derived from age-structured CSA estimates. Annual pregnancy denominators by district were calculated from CSA age–sex–fertility projections and apportioned to months using seasonal birth patterns.

### Data management and quality control

Duplicate rows were removed, harmonized district codes/names to the 166-district reference grid, and corrected date inconsistencies. Negative counts were set to missing. Completeness and internal consistency checks were performed monthly, comparing totals against district annual aggregates (within a ± 5% tolerance). Discrepancies were resolved through APHI with the district health office. The < 3% of missing district-months were excluded via complete-case analysis, and this is unlikely to introduce significant bias. To ensure robustness, primary analyses were conducted on a complete-case basis.

### Operational definitions

#### Resurgence

A district was classified as experiencing resurgence if cases in the main transmission season (September–December) of 2024 increased by ≥ 20% compared with the average of the same period in 2020–2023. This threshold was established through a structured survey of 28 purposively selected international, national, and regional experts from the Ministry of Health, Ethiopian Public Health Institute (EPHI), Amhara Public Health Institute (APHI), national and international academia, and partner organizations who have direct experience with the malaria programme (national and international expert opinion, 2024 unpublished data).

#### Endemic expansion

An increase in the number of districts exceeding the programme-defined endemic threshold (e.g., Annual Parasite Incidence (API) > 1 case per 1000 population). Hyperendemicity is defined as > 50 cases/1000 population/year [32].

### Statistical analysis

The analysis comprised descriptive statistics to summarize trends, Joinpoint regression to identify significant temporal changes, Auto Regressive Integrated Moving Average (ARIMA) modeling to characterize and forecast the test positivity rate, and spatial analyses to visualize distribution and identify clusters.

### Descriptive analysis

Annual and monthly malaria cases, Annual parasite incidence, species composition (*P. falciparum and P. vivax*), and test positivity rate (TPR) were summarized. The results were computed under the assumption of Poisson-distributed counts, and 95% confidence intervals (CIs) for the annual parasite incidence (API) were estimated using Byar’s approximation [36].

#### Temporal trend analyses

Joinpoint Regression (National Cancer Institute (NCI) v5.1.0) was used to identify inflection points in log-linear trends of monthly API [37]. Maximum 3 Joinpoints were permitted. Monte Carlo permutation tests (4500 iterations; α = 0.05) and Bayesian Information Criterion (BIC) were used to select the model [38]. The number and timing of Joinpoints, and Annual Percent Change (APC) with 95% CIs, where APC = (e^β – 1) × 100%.

#### Time-series analysis

Monthly TPRs were analyzed using seasonal ARIMA with a seasonal period of 12 (R v4.5.1; forecast v8.21; tseries). Stationarity was assessed via augmented Dickey-Fuller tests, and seasonal differencing (D = 1) was applied. Model selection was based on Akaike Information Criterion corrected/AICc/ and residual diagnostics, including Autocorrelation: ACF/PACF plots, Ljung–Box test (p > 0.05), normality: Q–Q plots, histograms, Homoscedasticity: residuals vs. fitted values plots (supplementary file).

### Spatial analysis and hotspot detection

District-level API was mapped for 2018 and 2024. Spatial clustering was analyzed using the Getis–Ord Gi* statistic with first-order queen contiguity and row-standardized weights. Statistical significance was assessed via 999 permutations with Benjamini–Hochberg FDR control (q = 0.05).

### Sensitivity analyses

#### Resurgence thresholds

Repeated classifications were performed using 15% and 30% thresholds; adjusted for population growth based on API-based baselines (supplementary file).

#### Species trends

Analyses were restricted to district-months with ≥ 50% (and ≥ 70%) microscopy use and repeated using only Pf/Pan RDT months. The *P. vivax* proportion over time was adjusted using logistic regression with diagnostic modality, RDT type, and district-level random intercepts (supplementary file).

### Software and reproducibility

Analyses used R (v4.5.1), NCI Joinpoint (v5.1.0), QGIS (v3.44.0), spdep/sf/sfdep (spatial), and forecast/tseries (time-series). Random seeds were fixed for permutation-based procedures. Code and the district crosswalk are available upon reasonable request to APHI, consistent with data-sharing policies**.**

### Ethical considerations.

The study received ethical approval from the Bahir Dar University College of Medicine & Health Sciences (Ref BDU-CMHS-IRB 3053/2024). Because the study used anonymized, aggregate surveillance data provided by the Amhara Public Health Institution, with no individual identifiers, individual consent was waived.

## Results

### Overall malaria burden (2014–2024)

**Fig. 2:**
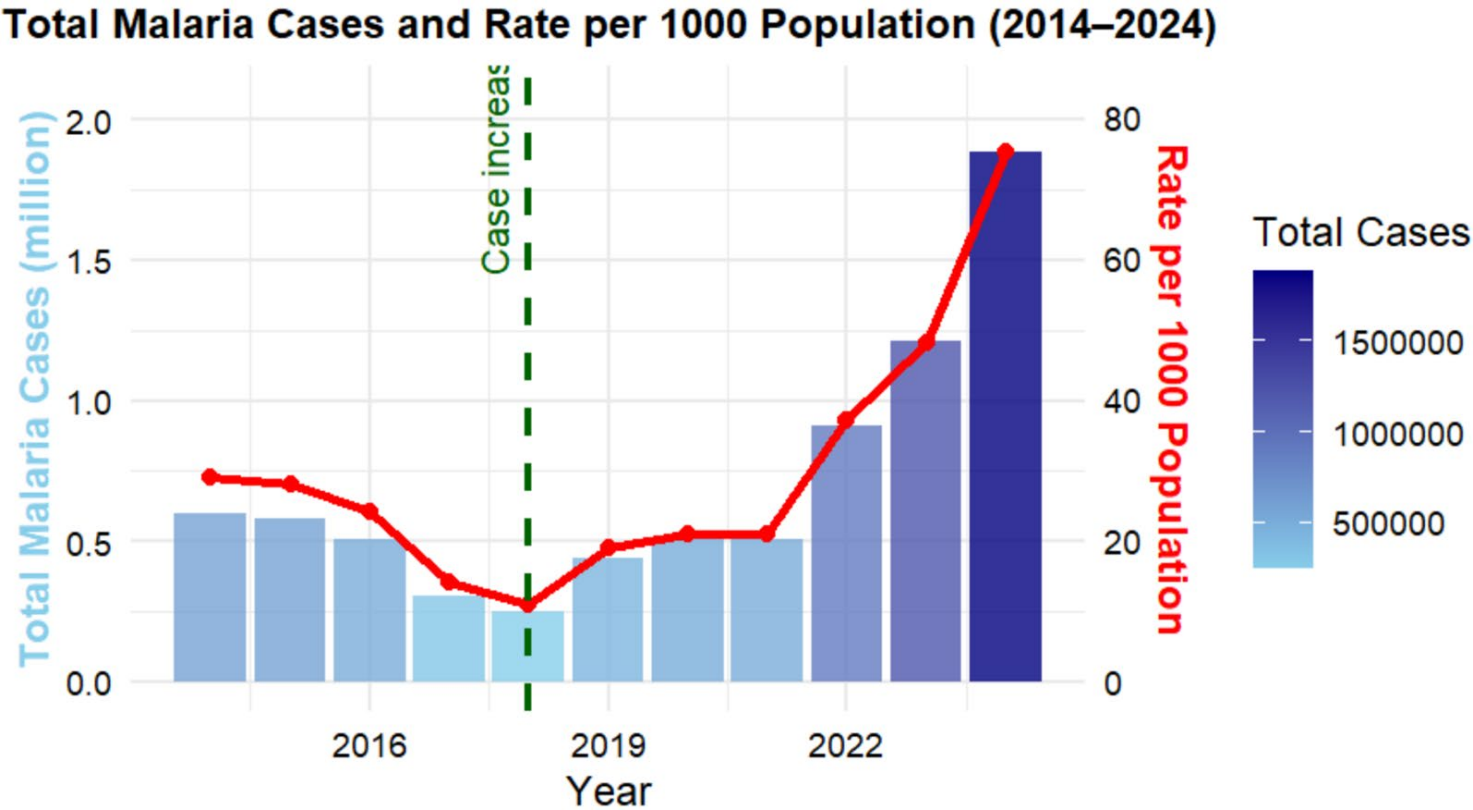
Trends in total annual malaria cases (columns) and incidence per 1000 population (line) in the Amhara Region, from January 2014 to December 2024

Over the 11 years, the Amhara Region reported a total of 7,710,733 malaria cases and 162 deaths. Annual cases declined from 597,064 in 2014 to around 247,960 in 2018, then increased to 1,884,802 in 2024, the highest level in the decade (Fig. 2). API followed a similar biphasic pattern: falling from 29.5 per 1000 population in 2014 to 10.9 per 1000 in 2018 (95% CI 10.85–10.93), then rising to 74.8 per 1000 in 2024 (95% CI 74.72–74.93). The 11-year mean API was 29.8 per 1000. Mortality remained low overall (mean rate 0.06 per 100,000), though deaths increased to 61 in 2024 (rate 0.24 per 100,000).

Inpatient malaria admissions declined from 11.7 per 100,000 in 2014 to 1.5 per 100,000 in 2018, then escalated to 23.3 per 100,000 in 2024. Similarly, malaria in pregnancy increased from 2.2 per 1000 pregnancies in 2018 to 25.6 per 1000 in 2024 (Supplementary file: Table S1).

### Seasonal patterns and monthly distribution

**Fig. 3:**
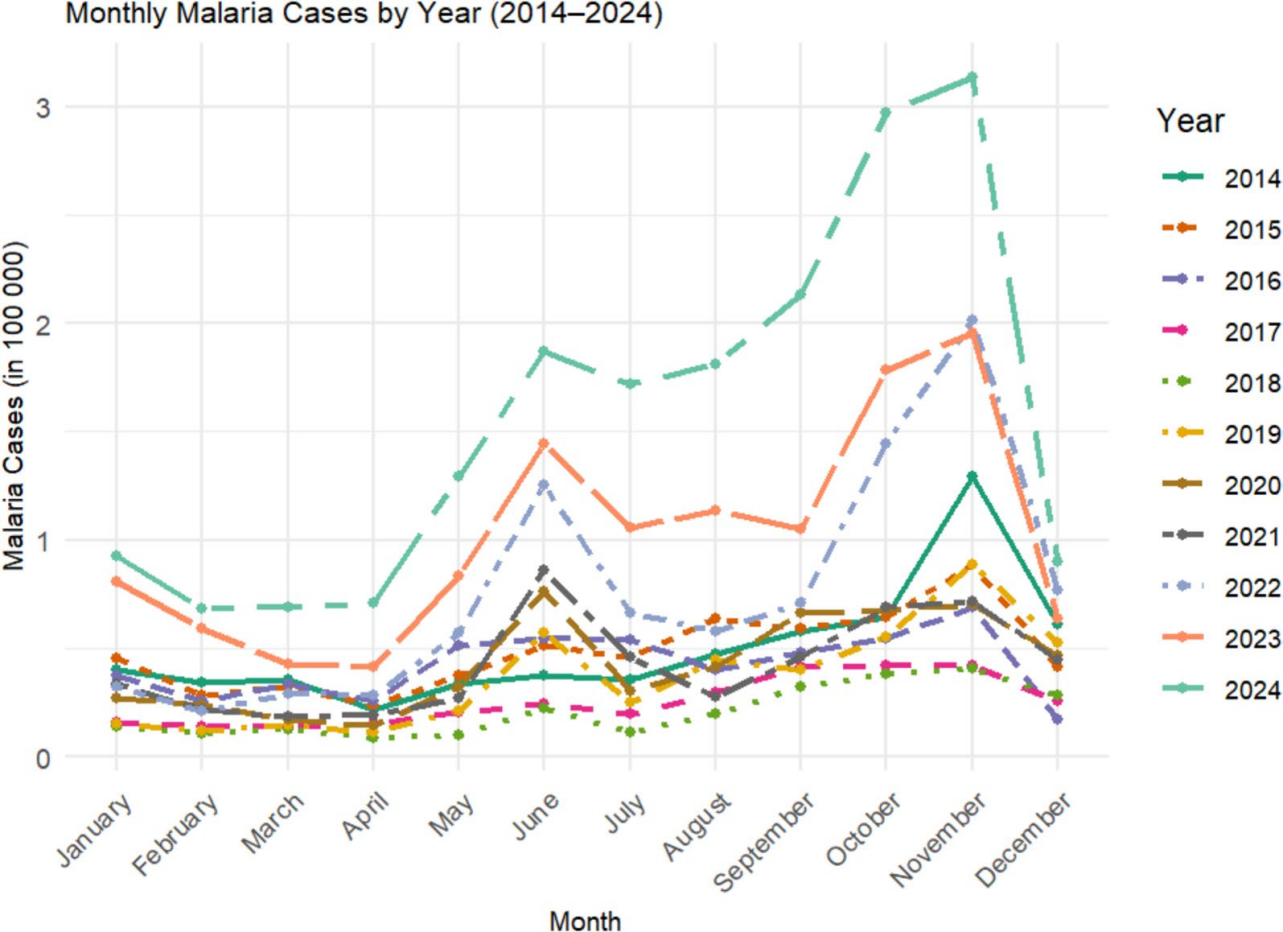
Seasonal pattern of monthly malaria cases in the Amhara Region, averaged across the low (2014–2018) and increased (2019–2024) phases, highlighting the intensification and broadening of transmission peaks

The monthly case distributions showed bimodal transmission peaks with a predominant post-rainy season peak (September–December) and a smaller April–July peak. During the increased phase (2019–2024), peaks intensified, shifted earlier by 4–6 weeks, and were accompanied by higher dry-season baselines compared with the low-transmission phase (2016–2018) (Fig. 3).

### Age-specific distribution

**Fig. 4:**
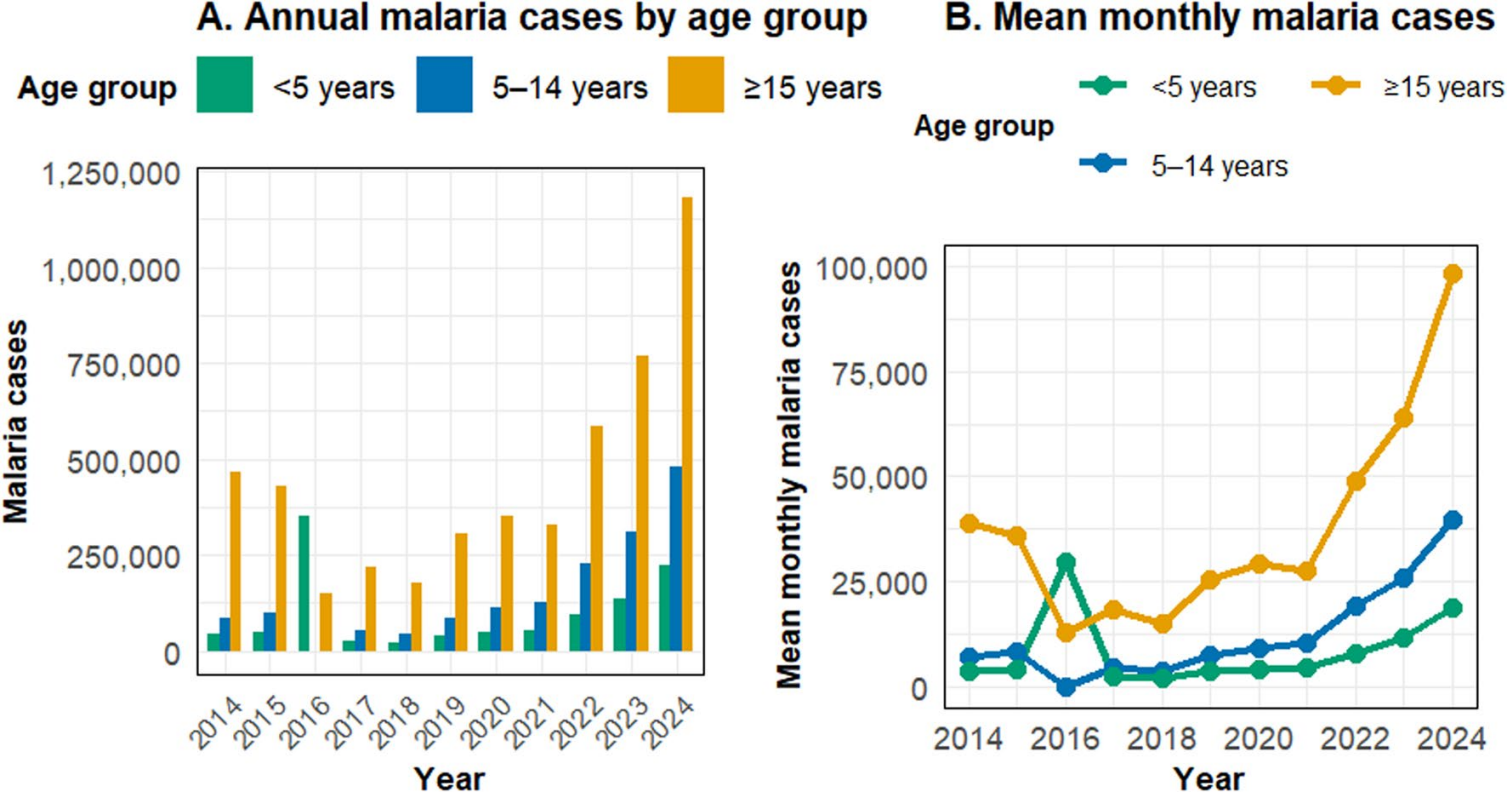
Annual malaria cases and mean monthly cases stratified by age group (under 5 years, 5–14 years, and 15  + years) in the Amhara Region, 2014–2024

Adults (≥ 15 years) accounted for 5,187,227 (62.3%) of total cases, with an 11-year average annual incidence of 41.6 per 1000 adults in this age group. Cases among children under 5 and those aged 5–14 years have also shown an annual increase. Among adults, the average annual number of cases was about 550,000 (± 50,000). For children aged 5–14 years, it was around 400,000 (± 40,000). Children under five had the lowest average, around 250,000 (± 30,000) (Fig. 4).

### Species distribution and shift

**Fig. 5:**
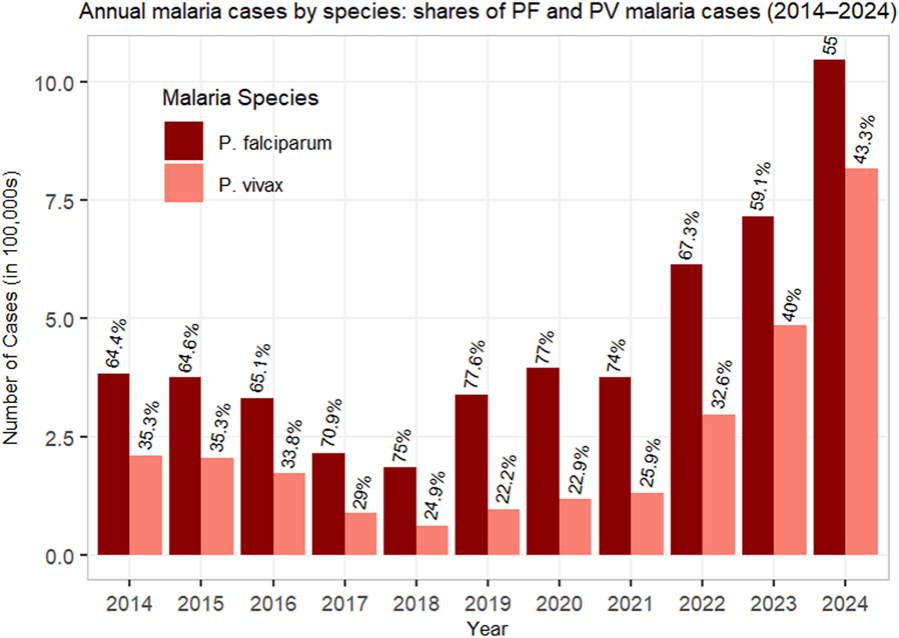
Species proportion of *P. falciparum* and *P. vivax* in the Amhara Region, 2014–2024

*Plasmodium falciparum* remained the dominant species throughout the study period (average incidence 19.7 per 1000). *Plasmodium vivax* incidence increased 11-fold, from 2.7 per 1000 in 2018 to 32.4 per 1000 in 2024. Consequently, the proportion of cases due to P. vivax rose from 24.9% in 2018 to 43% in 2024 (Fig. 5). Compound annual growth rates (2018–2024) were 51% for *P. vivax* and 31% for *P. falciparum*. The difference in species distribution over time was statistically significant (χ^2^ = 190,789.55, df = 10, p < 0.001).

**Fig. 6:**
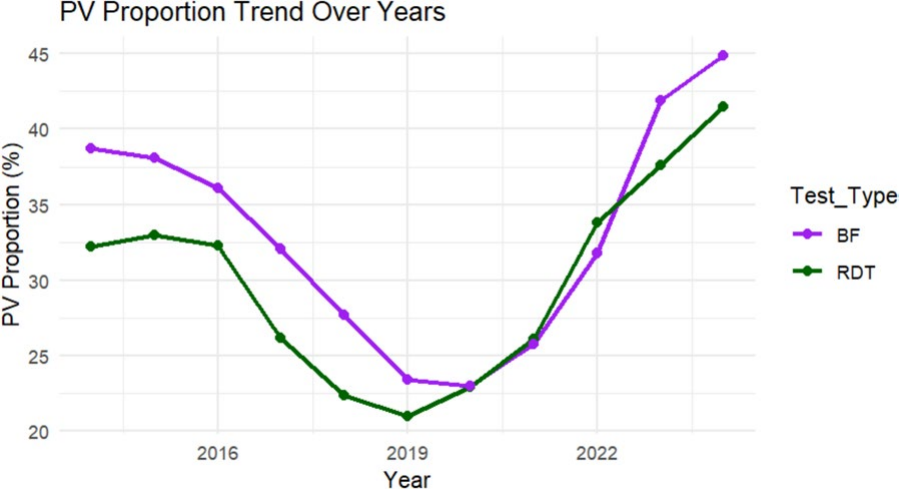
Proportion of *P. vivax* by diagnostic method (Microscope and RDT), Amhara region, 2014–2024

Rapid diagnostic tests (RDTs) surpassed microscopy as the primary detection method for *P. vivax* around 2019 (Fig. 6). Sensitivity analyses restricted to districts with ≥ 50% and ≥ 70% microscopy use yielded consistent findings. Random intercept models indicated substantial between-district heterogeneity in *P. vivax* contribution (Supplementary file: Table S2).

### Heatmap analysis of transmission dynamics

**Fig. 7:**
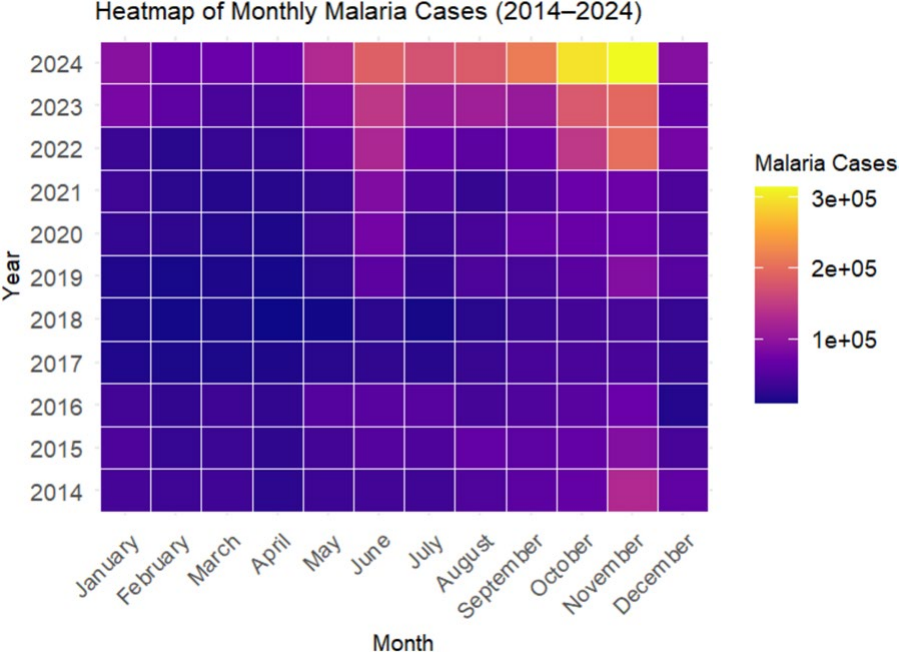
Heatmap of monthly malaria case counts from 2014 to 2024. Heatmap of monthly malaria case counts (log₁₀ scale), 2014–2024. Rows = years, columns = months. Dashed line marks late-2018 inflection. Colour gradient: dark blue (low, ~ 10^2^) to dark red (high, > 10^5^)

A monthly case heatmap revealed three patterns following late 2018 (Fig. 7): (i) Intensified post-rainy season peaks (August–November) after 2019; (ii) Prolonged high-transmission periods (≥ 5 months/year from 2022 onward); (iii) Elevated dry-season minima (January–March) compared with pre-2019 levels. Peak monthly case counts exceeded 300,000 in 2023–2024.

### Test positivity and seasonality

**Fig. 8:**
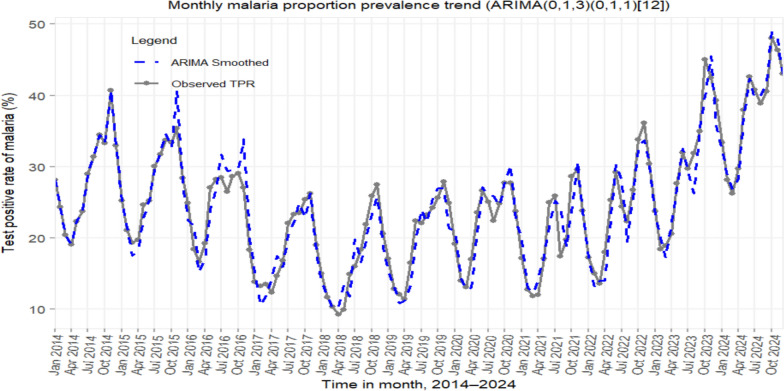
Trends in malaria test positivity rate (TPR) with ARIMA (0,1,3) (0,1,1) [12] model fit, Amhara Region, 2014–2024

TPR declined from 2014 to 2018, reaching a low of 10% in late 2018 (Fig. 8). A sustained increase began in 2019, accelerating after 2021. Seasonal fluctuations widened and became less predictable; peaks advanced by 4–6 weeks. By 2023–2024, TPR exceeded 50% for multiple consecutive months. For example, January TPR rose from 6.2% in 2018 to 32.1% in 2024.

The ARIMA (0,1,3) (0,1,1)[12] model fit the data well (AICc = 423.1; Ljung–Box Q = 8.3, p = 0.68), confirming robustness of trend estimates. Model assumptions were supported by residual diagnostics (Supplementary Fig. S1). In a train/test split (2014–2022 vs. 2023–2024), forecasts aligned closely with observed values. Results were consistent under the logit transformation of TPR (Supplementary Figs. S2, S3).

### Malaria resurgence: magnitude and spatial distribution

**Fig. 9:**
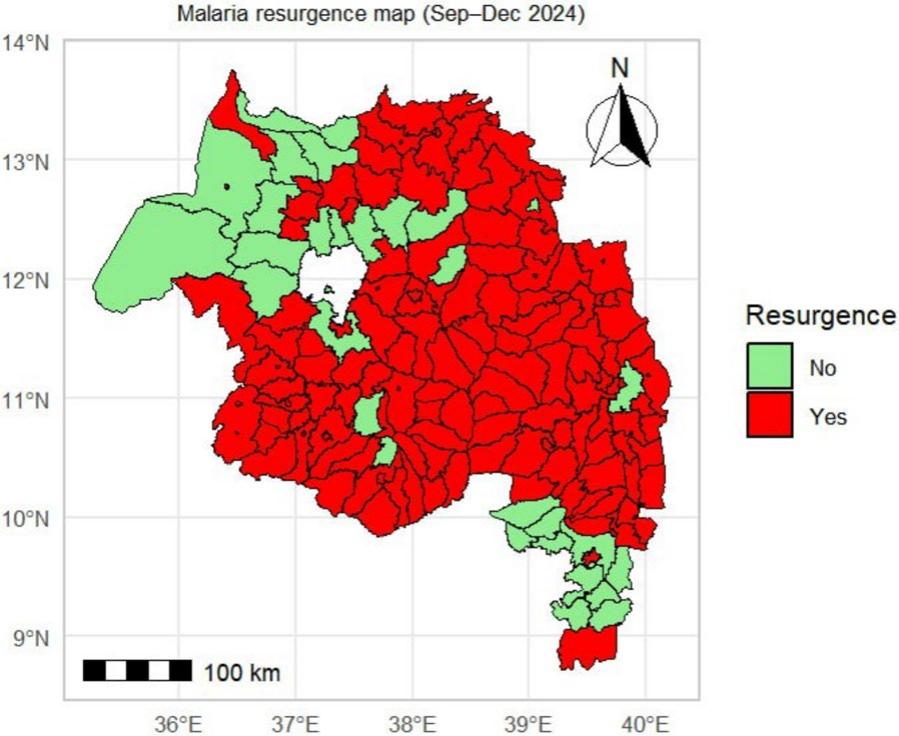
Spatial distribution of districts meeting the resurgence criterion (≥ 20% increase in cases during Sep–Dec 2024 compared to the 2021–2023 average for the same period) in the Amhara Region

Resurgence was most widespread in central, eastern, and southern zones. Non-resurgence districts were concentrated in the west, with scattered clusters in the north and centre (Fig. 9).

**Fig. 10:**
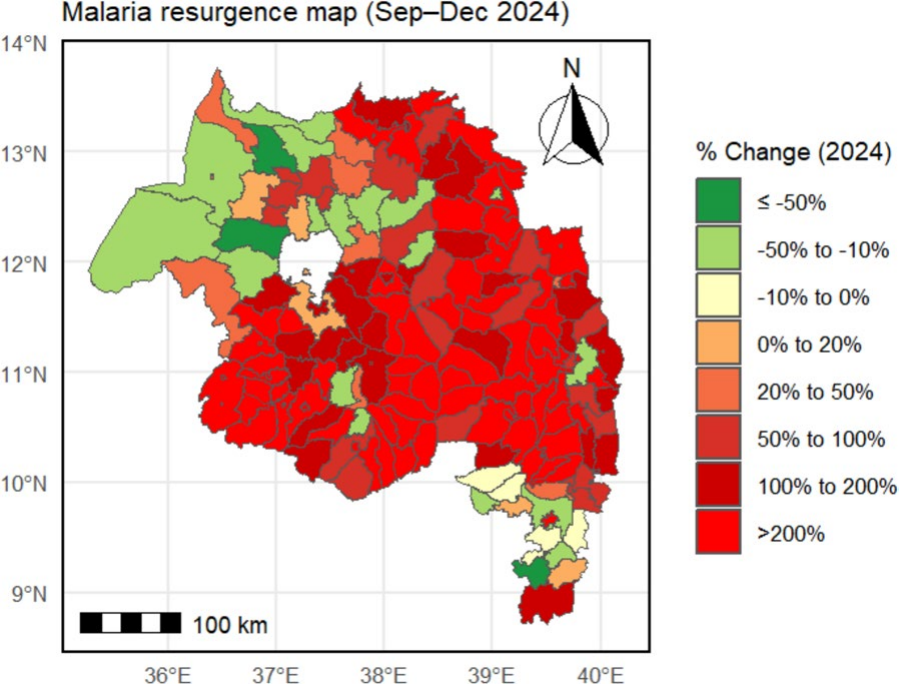
Magnitude of malaria resurgence, expressed as the percentage change in cases in Sep–Dec 2024 compared to the 2021–2023 baseline, for each district in the Amhara Region

By 2024, 137 of 166 districts (83%) met the resurgence criterion (≥ 20% increase in Sep–Dec 2024 cases vs. 2021–2023 baseline). Total regional cases during this period were 116% above baseline. Resurgence magnitude varied: 75 districts exceeded + 200%; 31 districts increased by + 100–200%. The largest increases occurred in Enemay (+ 1821%), Guangua (+ 1206%), and Ankesha (+ 1153%) (Fig. 10; Supplementary Table S2).

Classification was robust to alternative thresholds: near-perfect agreement was observed between the 20% threshold and 15% (Cohen’s κ = 0.956, p < 0.001) and 30% thresholds (κ = 0.938, p < 0.001) (Supplementary Figs. S5, S6).

### Malaria transmission hotspots

**Fig. 11:**
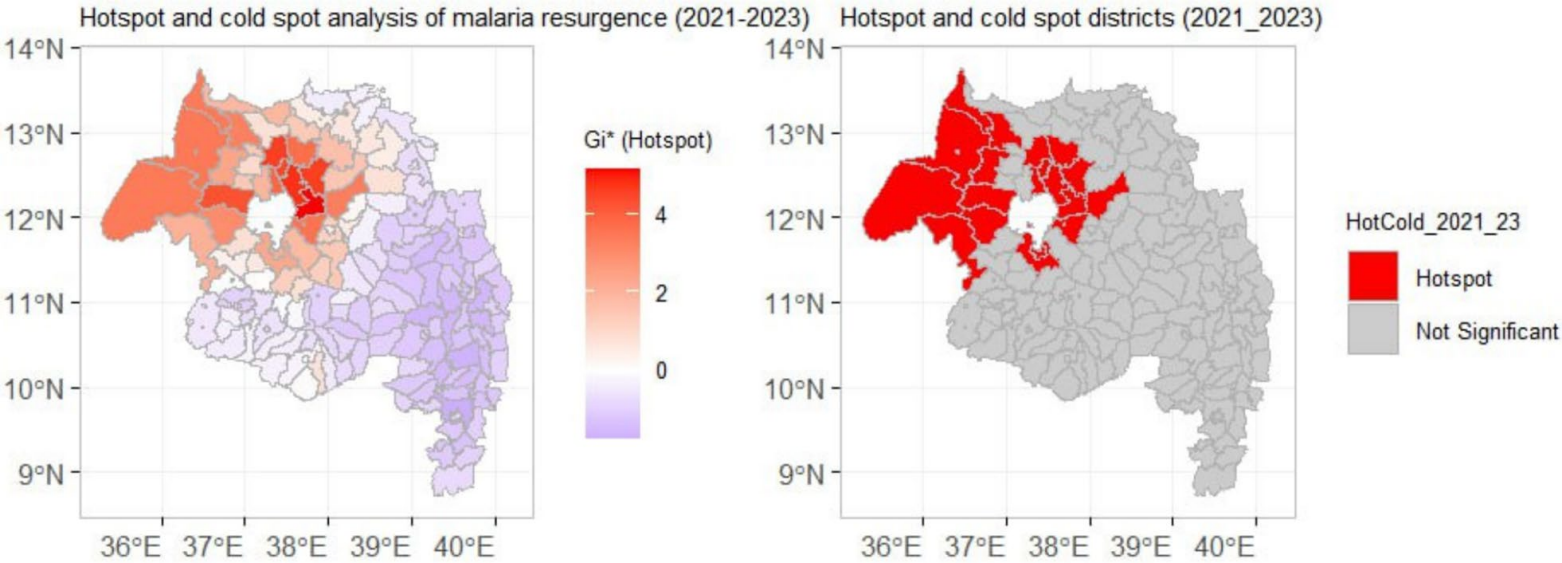
Spatial hotspot analysis of malaria incidence using the Getis-Ord Gi* statistic for the period 2021–2023. Hotspots (red) indicate clusters of districts with significantly higher incidence than expected, while cold spots (blue) indicate clusters with significantly lower incidence (p < 0.05, with FDR correction)

**Fig. 12:**
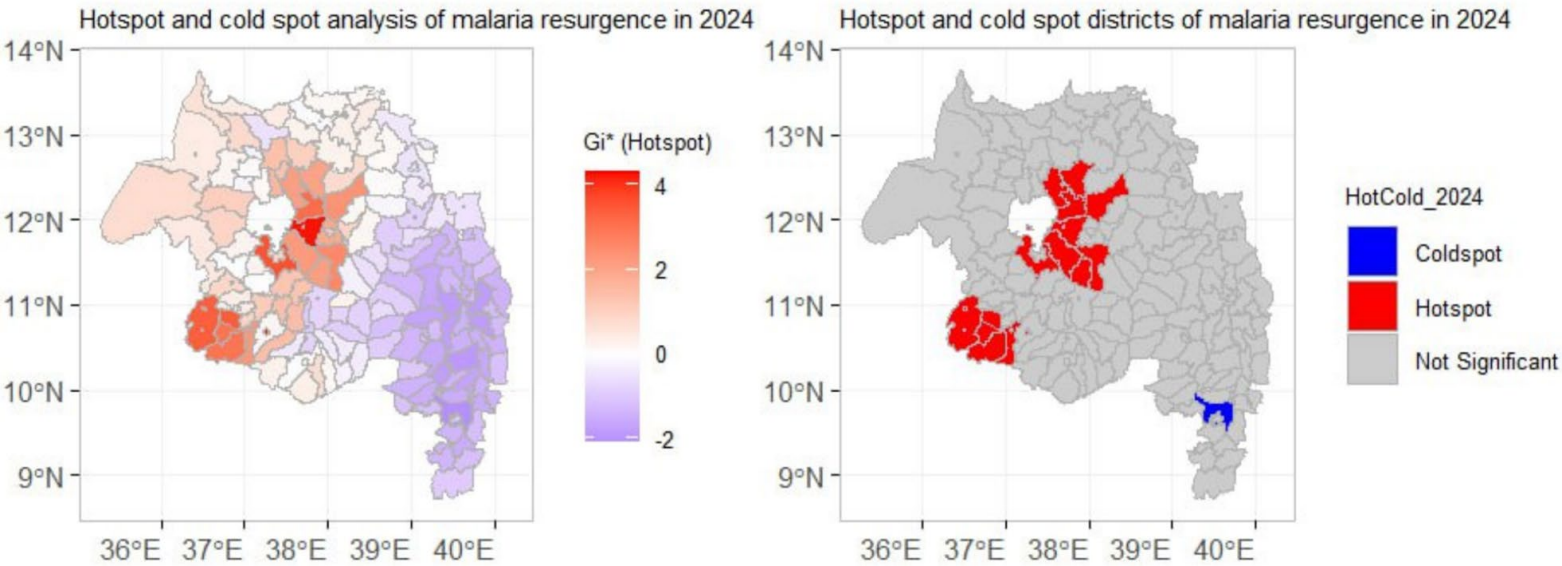
Spatial hotspot analysis of malaria incidence using the Getis-Ord Gi* statistic for the period 2024. Hotspots (red) indicate clusters of districts with significantly higher incidence than expected, while cold spots (blue) indicate clusters with significantly lower incidence (p < 0.05, with FDR correction)

Hotspot analyses of Empirical Bayes-smoothed API revealed localized high-confidence clusters in 2021–2023 (notably Dera and Farta). In 2024, the spatial pattern changed dramatically: isolated hotspots coalesced into an extensive, connected cluster of high-confidence hotspots covering most of the north-central area (e.g., North Mecha, Basoliben, Estie, South Achefer), while a separate cold-spot cluster persisted in the south (FDR q < 0.05) (Figs. 11 & 12).

### Spatiotemporal variation in malaria cases and TPR from 2018 to 2024

**Fig. 13:**
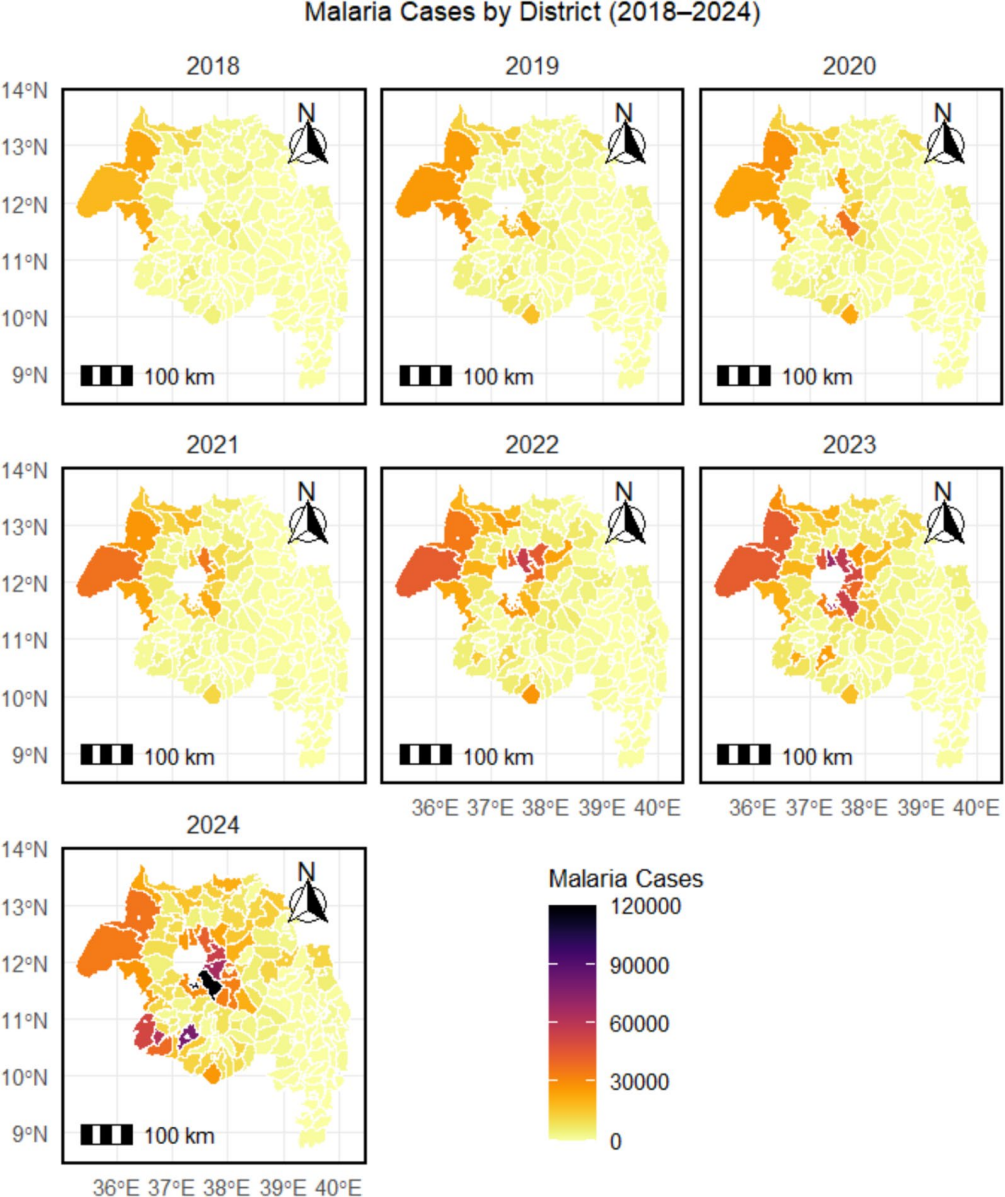
Choropleth maps depicting the spatiotemporal variation in malaria incidence (cases per 1000 population) at the district level for the years 2018 and 2024, showing marked geographical expansion

Comparison of 2018 and 2024 revealed marked geographic expansion. The number of endemic districts (API > 1 per 1000) increased by 78%. Peak district-level monthly caseload doubled from 60,000 to > 120,000. Urban centres such as Bahir Dar shifted from low burden (< 10,000 annual cases in 2018) to hyperendemic (> 90,000 annual cases in 2024). Highland districts that previously reported minimal transmission (e.g., 1200 cases in 2018) recorded > 40,000 cases in 2024 (Fig. 13).

**Fig. 14:**
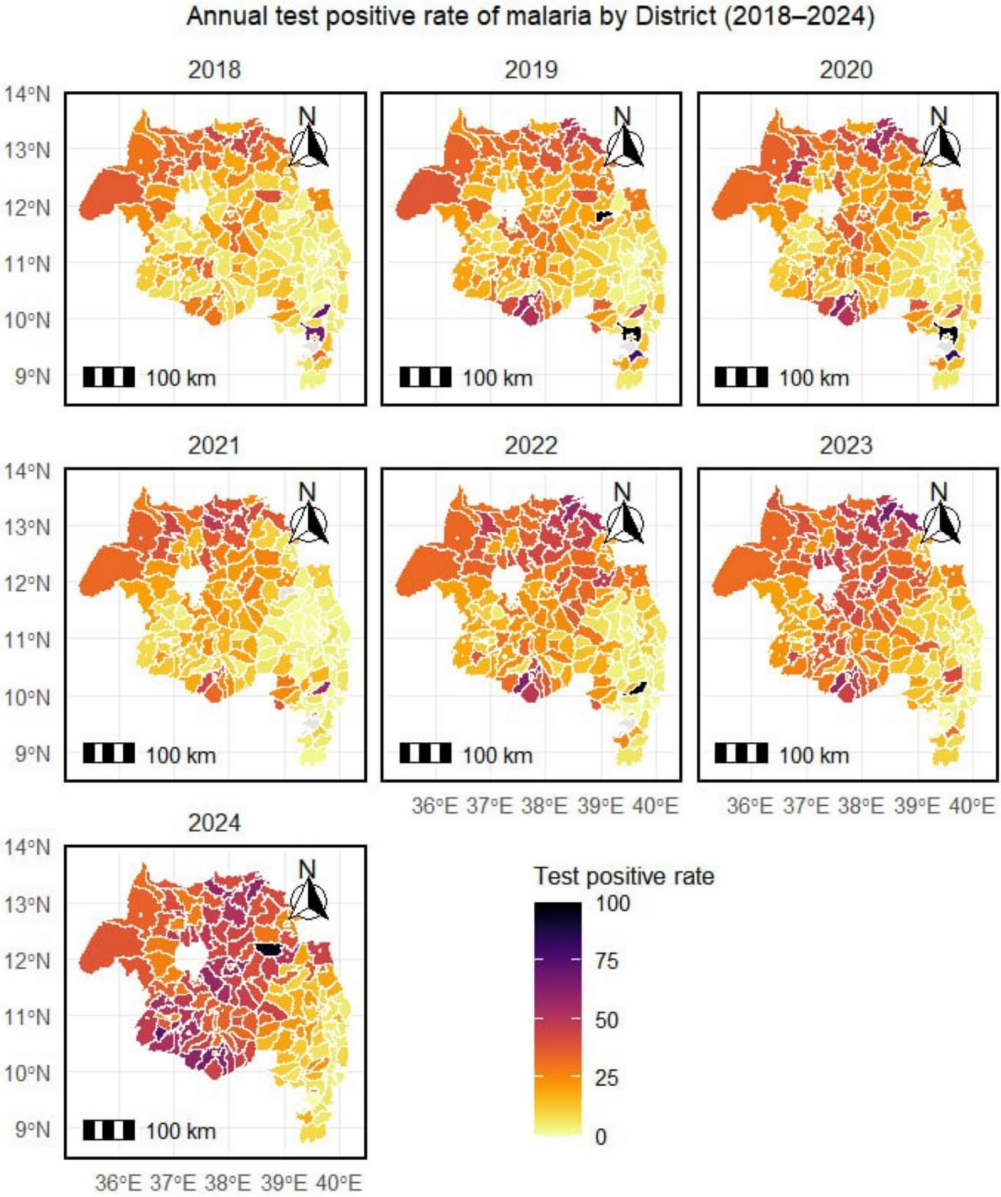
Choropleth maps depicting the spatiotemporal variation in malaria test positivity rate (TPR) at the district level for the years 2018 and 2024

Spatial mapping of TPR identified persistent high-TPR districts, particularly in central and western zones, where TPR ≥ 50% was sustained across multiple years (Fig. 14).

### Joinpoints regression analysis

**Fig. 15:**
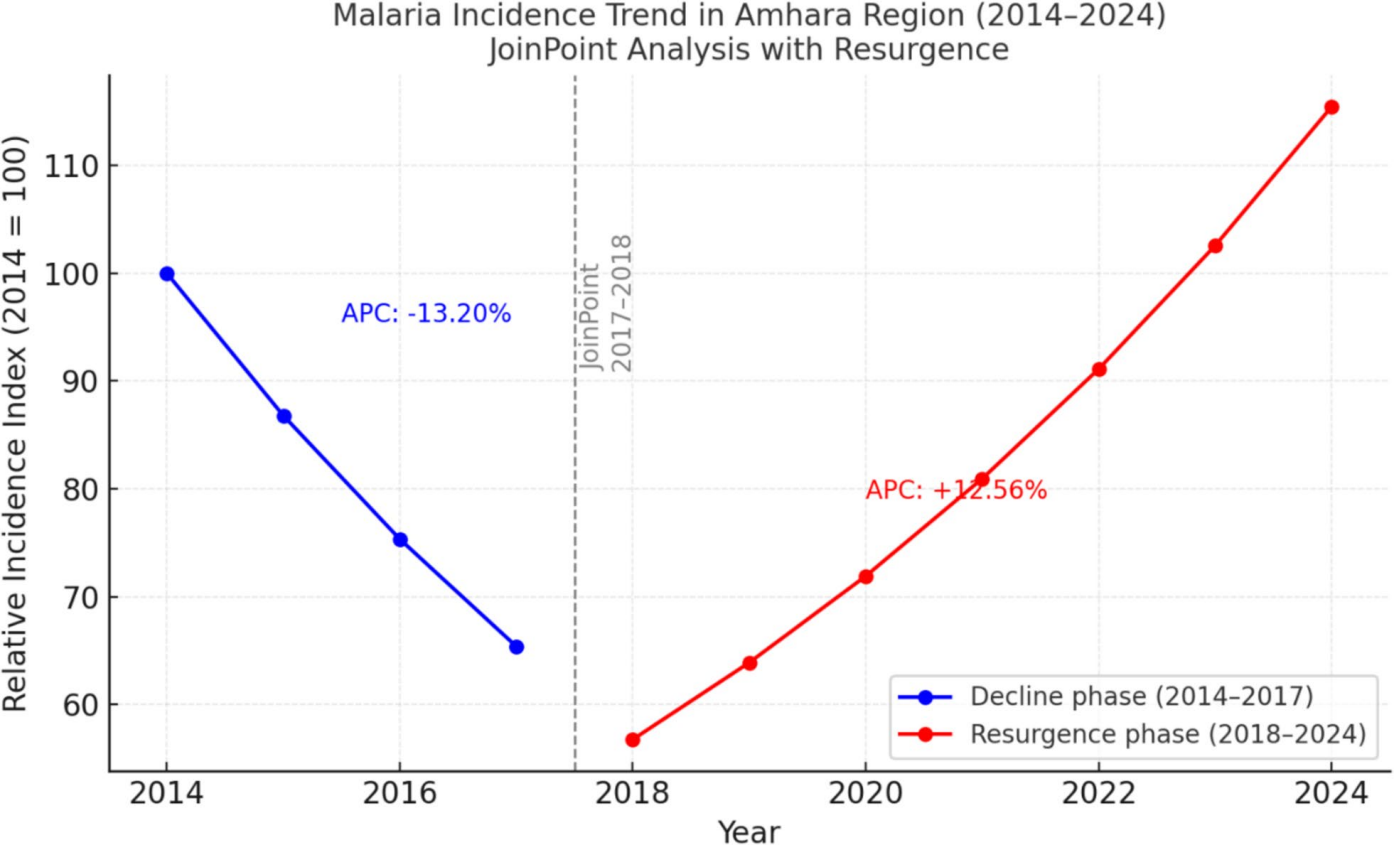
Joinpoint regression model showing trends in malaria incidence, Amhara Region, 2014–2024

Joinpoint regression identified a single inflection in late 2018 with two segments. From 2014 to 2017, incidence declined significantly at an annual rate of  − 13.2% (95% CI  − 27.28% to − 4.98%, p = 0.0008). This was followed by a significant increase from 2018 to 2024 at  + 12.56% per year (95% CI + 7.05% to + 22.95%, p < 0.000001), indicating a clear resurgence of malaria (Fig. 15, supplementary file: Table S4).

## Discussion

This 11-year analysis of over 7.7 million cases reveals a dramatic malaria resurgence in the Amhara region, with a clear inflection point in late 2018 that reversed earlier gains toward elimination [1, 3]. The resurgence features intensified and prolonged seasonality, expansion into urban and highland districts, and a marked rise in *P. vivax*.

This study described the initial decline (APC  − 13.2%, 2014–2017) coincided with the scale-up of ITNs/IRS/ACT [39–41], and was reflected in reduced admissions and malaria in pregnancy, consistent with global progress [42].

The study showed that the increasing resurgence trend (APC  + 12.6%, 2018–2024) is likely due to multiple factors, consistent with reports of the spread of the invasive mosquito *Anopheles stephensi*, which thrives in urban and highland areas [9–11]. This species thrives in artificial water containers common in urban settings and can transmit efficiently at higher altitudes, facilitating expansion into previously low-risk zones.

This resurgence was also likely exacerbated by regional conflicts that interrupted IRS and ITN campaigns and led to population displacement [12, 43], by evolving insecticide resistance [5, 15] and major disruptions to healthcare access and public health programmes during the COVID-19 pandemic [21, 44].

The study showed that the resurgence has changed geographically; endemic districts increased by 78%, with explosive growth in previously low-burden highlands and the transition of urban centres (e.g., Bahir Dar) to hyperendemic status. Hotspots consolidated into a contiguous north-central cluster by 2024, consistent with persistent source reservoirs that sustain regional transmission (Figs. 9, 10). This is a massive geographical extension of the transmission area.

The study revealed the pronounced rise in *P. vivax* (11-fold API increase; 43% of cases by 2024) complicates elimination. *Plasmodium vivax* relapses require radical cure, which is underused due to G6PD safety constraints [45]. Furthermore, the biological characteristics of *P. vivax*, including its wider temperature and gametocyte production, may facilitate its survival in reduced transmission settings and its expansion into new ecological niches like the highlands [46, 47].

The study revealed the increasing annual case counts among all age groups, with adults ≥ 15 years bearing the highest burden (62.3% of cases), underscoring a shifting demographic profile. This aligns with national surveillance trends [43] and highlights that malaria is not only a childhood disease in this region. This demographic shift underscores the critical need to expand surveillance, diagnosis, and prevention measures to include adult populations to achieve elimination objectives [19].

The study described the trend in test positivity rate (TPR) provides crucial insights into transmission intensity and health system performance. The temporary drop in TPR during the early phase of the COVID-19 pandemic (April–June 2020) likely reflects reduced healthcare access, a phenomenon reported across health programmes in Ethiopia [48]. The subsequent surge to a peak of 52.4% by 2024, coupled with the loss of dry-season suppression (e.g., January TPR rising from 6.2% in 2018 to 32.1% in 2024), indicates a profound increase in community-level transmission and a failure of control measures to interrupt transmission cycles completely. Forecast validation supports the persistence of high TPR without intensified control (Supplement file: Figs. S2–S3).

The study of spatiotemporal analysis showed that the spatiotemporal expansion of malaria, particularly an increase in the highland burden, might be consistent with the known temperature suitability for the establishment of invasive vectors like *An. stephensi* [49]. Likewise, the heavy urban clustering also corresponds with environmental factors such as water storage practices that create potential breeding sites, which have been linked to malaria risk in urban Ethiopian settings [11, 50]. These patterns are also consistent with broader models of how climate change and land use change can facilitate malaria transmission in previously unsuitable areas [51].

Spatial hotspot analysis revealed the consolidation of historically distinct hotspots in the north-central region into a single expansive and contiguous cluster by 2024 (Figs. 11, 12). This suggests the formation of a persistent transmission reservoir that can continually fuel regional epidemics, aligning with the concept of transmission "hotspots" that are critical to sustaining malaria parasitaemia even amidst broader control efforts [52, 53]. Targeting such reservoirs with coordinated, cross-district interventions is a cornerstone of malaria elimination strategy [54].

Joinpoint analysis confirmed a significant trend reversal in late 2018. The resurgence rate approximated the earlier decline rate, highlighting systemic vulnerability as programmes approach elimination, a recognized “elimination paradox” where prior success leads to complacency and heightened rebound risk when vulnerabilities converge [19, 55].

Highland districts, while reporting lower absolute case counts, exhibited high TPR indicative of low population immunity and efficient focal transmission [56]. This underscores the need for highland-optimized surveillance (rapid detection and response), even when case counts appear low [57, 58]. This underscores that highland regions are highly susceptible to epidemics and require specific surveillance systems optimized for rapid outbreak detection and response, despite a perceived low risk based on case counts alone [16].

### Strengths and limitations

This study’s strengths include an 11-year, region-wide dataset covering 166 districts, and the use of advanced statistical/spatial tools (Joinpoint, ARIMA, hotspot analysis). Limitations include a lack of district-level intervention data (ITN/IRS coverage), entomology, climate, and conflict, which may confound observed patterns, and excluding referral hospital cases.

## Conclusion

This study provides evidence of malaria resurgence in the Amhara Region of Ethiopia, reversing the gains of the past decade. Eleven years of surveillance data reveal a concerning epidemiological shift marked by three critical changes: a temporal increase in incidence with a turning point in late 2018, a biological shift toward greater prevalence of the resilient *P. vivax* parasite, and geographical expansion into urban and highland settings previously considered low risk.

These findings illustrate an “elimination–resurgence paradox”: prior successes may have reduced operational readiness and masked systemic vulnerabilities, leaving programmes unprepared for converging threats, including invasive vectors, conflict, climate variability, and diagnostic gaps.

To reverse this trend, a fundamental strategic shift is required. Current approaches are insufficient; interventions must be stratified and adaptive. Immediate scale-up of G6PD testing and radical cure for *P. vivax*, coupled with geographically targeted vector control in urban and highland hotspots, enhanced surveillance in highland and urban areas, and innovative measures against urban vectors, is essential to avert a regional malaria burden.

## Supplementary Information

Supplementary material 1.

## Data Availability

Aggregated surveillance data are available from APHI on reasonable request, subject to data-sharing policies.

## Abbreviations

ACT: Artemisinin-based combination therapy
APC: Annual Percent Change
APHI: Amhara Public Health Institute
ARIMA: Autoregressive integrated moving average
CAGR: Simple Compound Annual Growth Rate
G6PD: Glucose-6-phosphate dehydrogenase
IRS: Indoor residual spraying
ITN: Insecticide-treated net
TPR: Test positivity rate
