# Supplementary material for: Resurgence of malaria in the Amhara Region, Ethiopia (2014–2024): trends, spatial expansion, and control challenges
Source: Malar J. 2025 Nov 25;24:425. doi: 10.1186/s12936-025-05668-0 (PMC12645681; doi:10.1186/s12936-025-05668-0)
Supplement: Supplementary file 1 — Supplementary material 1. [file 12936_2025_5668_MOESM1_ESM.docx]

**Supplementary Files**

Supplementary Figures


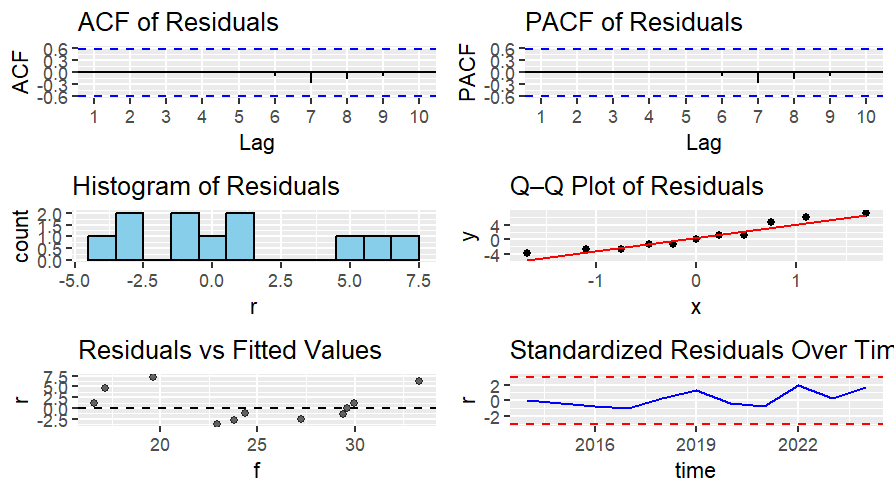


Fig. S1. ARIMA(0,1,3) model fitted to the malaria test positivity rate (TPR) series, 2014–2024

Diagnostics for the ARIMA (0,1,3)(0,1,1)[12] model fitted to the malaria test positivity rate (TPR) series. **(A)** Actual TPR values (black line) with model fit (red line) and forecasts. **(B)** ACF and **(C)** PACF of model residuals show no significant autocorrelations. **(D)** A Ljung-Box test (p = 0.68) confirms residual independence. **(E)** A Q-Q plot and **(F)** a histogram show that residuals are approximately normally distributed. **(G)** A plot of residuals vs. fitted values shows no evidence of heteroscedasticity. Collectively, these diagnostics indicate that the model adequately captures the structure in the TPR data and fulfills its assumptions.


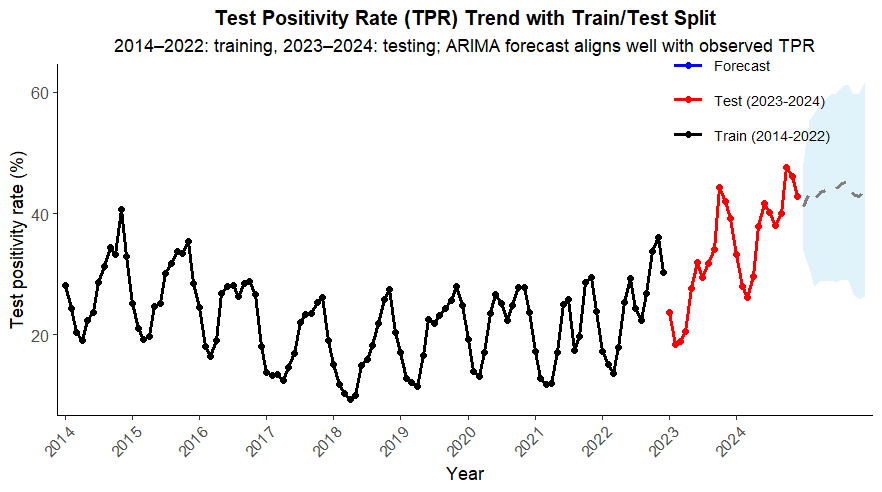


Fig. S2. Trend of Test positivity rate with train and test split; (2014–2022 vs 2023–2024), Amhara Region.


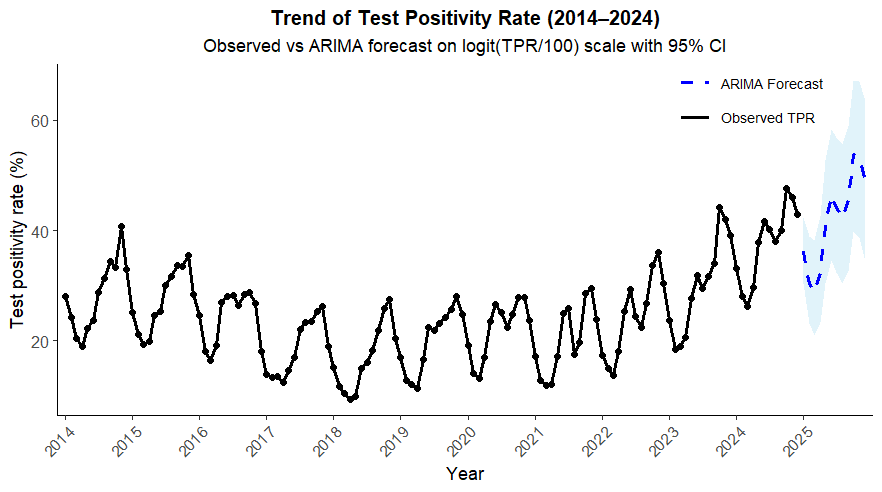


Fig. S3. Trends of TPR under a logit (TPR/100) transformation, 2014 to 2024


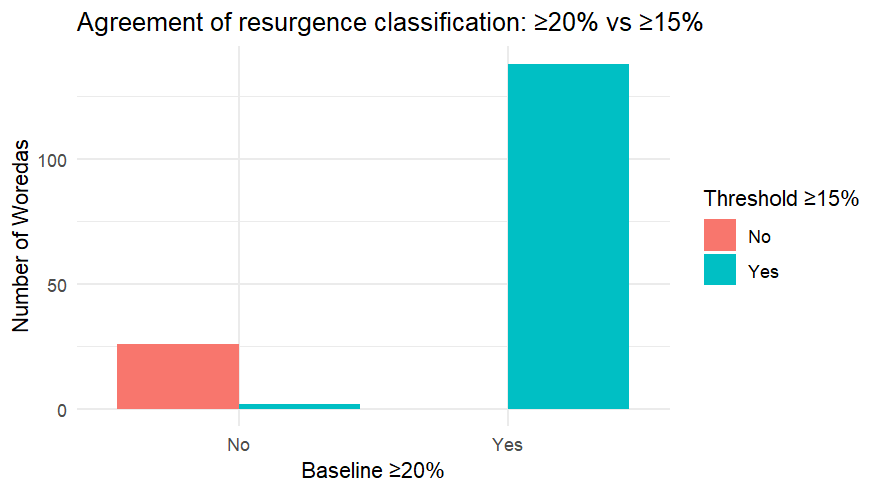


Fig. S4. Agreement of resurgence classification: Resurgence ≥20% (Base line) vs. ≥ 15%


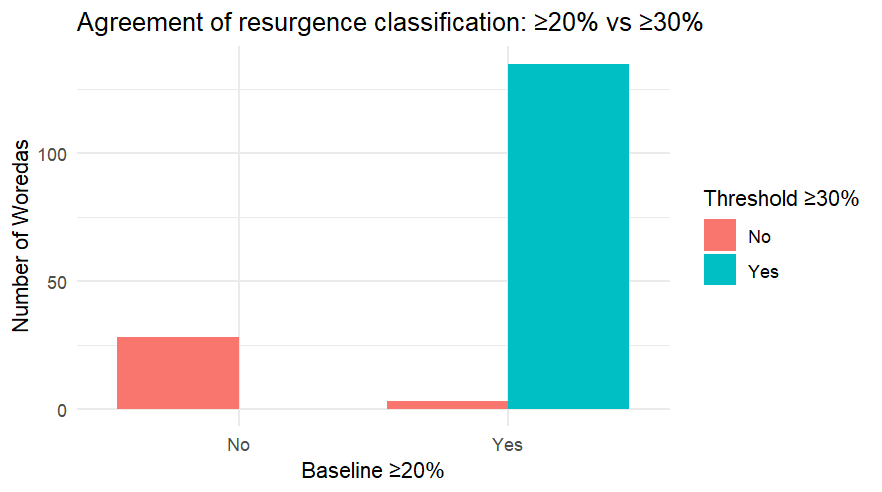


Fig. S5. Agreement of resurgence classification: Resurgence ≥20% (Base line) vs. ≥ 30%

**Supplementary Tables**

Table S 1. Annual malaria indicators, Amhara Region, 2014–2024.

| Year | API/1000 population | Deaths per 100,000 population | Inpatient admissions per 100,000 population | Malaria in pregnancy per 1000 pregnancies | *P. vivax incidence per 1,000 population* | *P. falciparum incidence per 1,000 population* |
| --- | --- | --- | --- | --- | --- | --- |
| 2014 | 29.5 (29.4–29.6) | 0.09 | 11.7 | 6.1 | 10.4 | 19 |
| 2015 | 28.1 (28.0–28.2) | 0.06 | 4.6 | 5.2 | 9.9 | 18.1 |
| 2016 | 23.8 (23.7–23.9) | 0.03 | 4.3 | 3.8 | 8 | 15.5 |
| 2017 | 14.1 (14.0–14.2) | 0.01 | 2.5 | 2.6 | 4.1 | 10 |
| 2018 | 10.9 (10.8–11.0) | 0.01 | 1.5 | 2.2 | 2.7 | 8.2 |
| 2019 | 18.8 (18.7–18.9) | 0.04 | 3.3 | 3.9 | 4.2 | 14.6 |
| 2020 | 21.2 (21.1–21.3) | 0.02 | 3.9 | 5.0 | 4.9 | 16.3 |
| 2021 | 21.0 (20.9–21.1) | 0.02 | 2.1 | 5.7 | 5.4 | 15.5 |
| 2022 | 37.2 (37.1–37.3) | 0.09 | 6.1 | 8.7 | 12.1 | 25 |
| 2023 | 48.0 (47.9–48.1) | 0.06 | 9.6 | 12.9 | 19.2 | 28.3 |
| 2024 | 74.8 (74.7–74.9) | 0.24 | 23.3 | 25.6 | 32.4 | 41.6 |
| Average | 29.5 (29.4–29.6) | 0.06 | 6.8 | 7.7 | 10.6 | 19.7 |

Footnotes: API = confirmed malaria cases per 1,000 population; denominators for pregnancy estimates from Central Statistical Agency (CSA) projections; CFR = case fatality rate; *OR: Odds Ratio; CI: Confidence Interval.*

Table S2. Mixed-effects logistic regression models for P. vivax among malaria positives

| Model | Predictor | OR | CI_lower | CI_upper | p_value |
| --- | --- | --- | --- | --- | --- |
| Microscopy ≥50% | Month (1–12) | 0.97 | 0.96 | 0.97 | <0.001 |
| Microscopy ≥50% | RDT vs Microscopy | 0.85 | 0.83 | 0.86 | <0.001 |
| Microscopy ≥70% | Month (1–12) | 0.97 | 0.96 | 0.98 | <0.001 |
| Microscopy ≥70% | RDT vs Microscopy | 0.83 | 0.81 | 0.85 | <0.001 |
| All data (adjusted) | Month (1–12) | 0.97 | 0.97 | 0.97 | <0.001 |
| All data (adjusted) | RDT vs Microscopy | 0.84 | 0.83 | 0.85 | <0.001 |
| AIC BIC logLik -2*log(L) df.resid  557983.6 558099.7 -278977.8 557955.6 29434 | | | | | |
| Random effects:  Groups Name Variance Std.Dev.  District (Intercept 0.2891 0.5377  Number of obs: 29448, groups: District, 166 | | | | | |

Table S3. Top 20 districts showing the highest resurgence (% change from 3-year baseline), Amhara Region, 2024.

| Zone | Woreda | ADM3_PCODE.x | Resurgence changes in % | Classification |
| --- | --- | --- | --- | --- |
| East Gojjam | Enemay | ET030606 | 1821 | Yes |
| Awi | Guangua | ET030904 | 1205. | Yes |
| Awi | Ankasha | ET030903 | 1154 | Yes |
| Wag Hemra | Gaz Gibla | ET030804 | 1101.2 | Yes |
| South Wollo | Ambasel | ET030404 | 1062.5 | Yes |
| North Wollo | Wadla | ET030305 | 934.8 | Yes |
| Awi | Zigem | ET030997 | 881.2 | Yes |
| North Shewa (AM) | Gishe Rabel | ET030506 | 856.6 | Yes |
| North Wollo | Kobo Town | ET030399 | 842.2 | Yes |
| North Wollo | Gazo | ET030312 | 836 | Yes |
| South Wollo | Legehida | ET030420 | 810.5 | Yes |
| East Gojjam | Sedae | ET030619 | 782.7 | Yes |
| East Gojjam | Dejen | ET030613 | 782.1 | Yes |
| West Gojjam | Wemberma | ET030711 | 777.2 | Yes |
| South Wollo | Were Ilu | ET030415 | 758.4 | Yes |
| Awi | Injibara Town | ET030996 | 753.4 | Yes |
| East Gojjam | Michakel | ET030609 | 743.4 | Yes |
| West Gojjam | Bure (Am) | ET030710 | 688.2 | Yes |
| South Wollo | Albuko | ET030408 | 679.2 | Yes |
| Awi | Ayehu Guwagusa | ET030908 | 668.9 | Yes |

Table S4. Annual percent change (APC) in malaria incidence from Joinpoint regression, Amhara Region, 2014–2024.

| Segment | Segment Period | APC | 95% CI | *p-Value |
| --- | --- | --- | --- | --- |
| 1 | 2014–2017 | -13.20%* | -27.28% to -4.98% | 0.0008 |
| 2 | 2018–2024 | +12.56%* | +7.05% to +22.95% | <0.000001 |

*Statistically significant (α=0.05).
